# Sex-specific maternal effects in a viviparous fish

**DOI:** 10.1098/rsbl.2015.0472

**Published:** 2015-08

**Authors:** Loeske E. B. Kruuk, Julianne Livingston, Andrew Kahn, Michael D. Jennions

**Affiliations:** 1 Research School of Biology, The Australian National University, Canberra, Australian Capital Territory 0200, Australia; 2 School of Biological Sciences, University of Edinburgh, Edinburgh EH9 3FL, UK

**Keywords:** maternal effects correlation, *Gambusia*, offspring size, development rate, life-history trade-offs

## Abstract

Mothers vary in their effects on their offspring, but studies of variation in maternal effects rarely ask whether differences between mothers are consistent for sons and daughters. Here, we analysed maternal effects in the mosquitofish *Gambusia holbrooki* for development time and adult size of sons and daughters, and a primary male sexual character (gonopodium length). We found substantial maternal effects on all traits, most notably for gonopodium length. There were significant correlations within each sex for maternal effects on different traits, indicative of trade-offs between development rate and adult size. By contrast, there was no evidence of any consistency in maternal effects on sons and daughters. This suggests that the evolution of maternal effects will follow independent trajectories dependent on sex-specific selection on offspring. Importantly, failure to recognize the sex-specific nature of maternal effects in this population would have substantially underestimated the extent of their variation between mothers.

## 1. Introduction

What makes a high-quality mother? Maternal investment can play a critical role in determining an offspring's phenotype and hence fitness, thereby making it an important evolutionary adaptation [1–3]. However, quantifying maternal performance is complicated if mothers do not invest equally in all offspring. In particular, maternal investment into sons and daughters can vary, for example by variation in the offspring sex ratio [4], or by subsequent differential investment into sons and daughters [5]. If sons and daughters have different requirements, some mothers might be better at producing daughters and others at producing sons. Alternatively, if there is high variation in maternal resource acquisition, do some mothers produce better daughters *and* better sons?

Maternal effects are the impact of a mother on her offspring in addition to direct effects of inherited genes. It is possible to compare maternal effects on offspring of either sex, and therefore to quantify the consistency of maternal effects on sons and daughters: for example, maternal effects on immune defence in side-blotched lizards have a strongly negative correlation across the sexes [6], whereas measures of annual reproductive success show positive covariance in maternal effects in red deer ([7], though these could potentially be due to shared common-environment effects rather than maternal investment *per se*). However, in general, estimates of these cross-sex maternal effect correlations are rare.

We also know surprisingly little about how maternal effects on one trait relate to those on another (though see [8]), nor if there are detectable maternal trade-offs (i.e. negative relationships between maternal effects on different traits). For example, do maternal effects mediate the frequently observed phenotypic trade-off between development rate and size at maturity [9]? Finally, although sexual selection seemingly plays a critical role in determining parental care [10], the converse role of maternal effects on sexually selected traits has received little attention [11–13]—possibly because of the expectation that, at least in long-lived animals, maternal effects fade with age and are unlikely to affect adult sexual traits (e.g. [14,15]).

Here, we present data on maternal effects on maturation rate and adult body size in both sons and daughters, and on a sexually selected male trait, in the mosquitofish *Gambusia holbrooki*. There is evidence in this species for adaptive sex allocation in the form of seasonal sex ratio changes [16], and for sex differences in response to low food availability [17]. We ask: (i) how important are maternal effects in determining phenotypic variance between individuals, compared to the contribution of heritable genetic effects; (ii) how do maternal effects covary across traits; and, crucially, (iii) how consistent are maternal effects across daughters and sons?

## 2. Material and methods

### (a) Study species, breeding design and traits

The mosquitofish *G. holbrooki* is a poeciliid fish endemic to North America, but now a hyper-abundant pest species in Australia [18]. Fertilization is internal and males transfer sperm via a modified anal fin (gonopodium). We used a standard full/half-sib breeding design in which 69 virgin dams produced viable offspring from 19 sires. Approximately nine offspring per dam were then reared individually, under either normal or restricted (days 7–28) food conditions [17]. Here, our analysis focused on sources of variation in five adult traits: *body length* (snout to base of caudal fin, in mm) and *age at sexual maturity* (in days)*,* for both sexes (*N* = 297 females, 303 males); and male *gonopodium length* (apical tip to base, in millimetres; *N* = 261 males). Further methodological details and summary statistics are in the electronic supplementary material, table S1.

### (b) Statistical analyses

We fitted multivariate mixed models to the five traits in *ASReml-R* [19]. All traits were first standardized to unit variance, and phenotypic (co)variances are shown in the electronic supplementary material, table S1. We then quantified components of (co)variance using a multivariate ‘animal model’, with random effects of an additive genetic effect (with covariance structure defined by relatedness between individuals) and a maternal effect (grouping individuals by mother, [20]). The fixed effects were food treatment (two levels) and shelf row (10 levels, to represent shelves at different heights; see the electronic supplementary material for details).

Multivariate (or ‘multi-response’) mixed models allow covariances and correlations between traits to be estimated for each specified random effect. At the phenotypic level, there cannot be correlations between male and female traits as they occur in different individuals. However, at the additive genetic or maternal effects level, a multivariate model can estimate cross-sex correlations: for example, a positive cross-sex maternal effects correlation for size indicates that mothers who produce larger daughters have larger sons. Similarly, the model quantifies cross-sex correlations between traits (e.g. whether mothers with large daughters have fast-developing sons). The significance of (co)variance components was tested using likelihood-ratio tests, and given the multiple testing involved we adopt a significance criterion of *p* < 0.01.

## 3. Results

Maternal effects were consistent across food treatments (see the electronic supplementary material) and explained a significant proportion (28–52%) of the variation in all five adult traits (table 1, maternal effects). However, we found no evidence of significant additive genetic variance for any trait (table 1, additive genetic effects), so we did not fit genetic covariances between traits.

**Table 1.** Variance–covariance matrices from the multivariate model of adult length and age at sexual maturity in both sexes and male gonopodium length. The three 5 × 5 matrices give (co)variance components for maternal, additive genetic and residual effects, respectively. Diagonal cells of each contain variances and proportions (both in italics), below-diagonal cells contain covariances, above-diagonal contain correlations (SEs in brackets). Traits were all standardized to unit variance; variance components can therefore be compared approximately across traits. Covariance/correlation cells are blank for additive genetic effects, because there were no significant variance components, and for cross-sex residual components. Light grey shading indicates within-sex components of (co)variance.

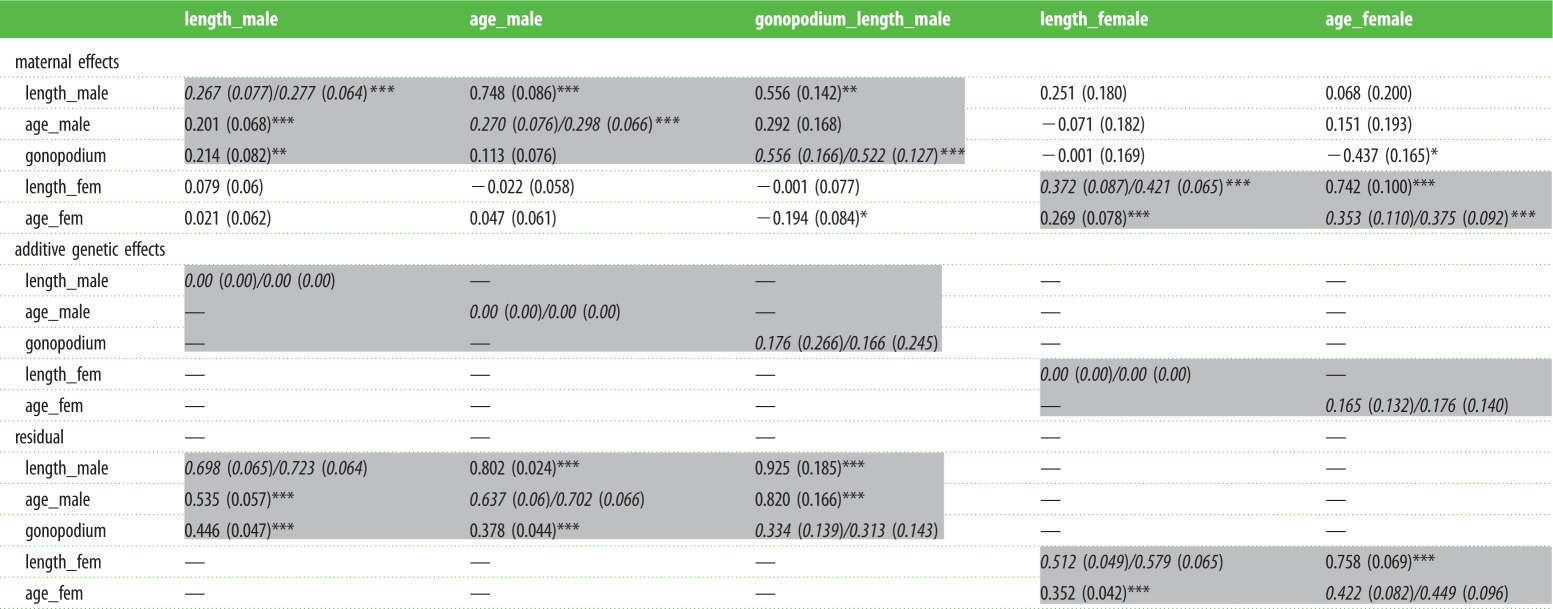

*p < 0.05, **p < 0.01, ***p < 0.001.

**Figure 1.**
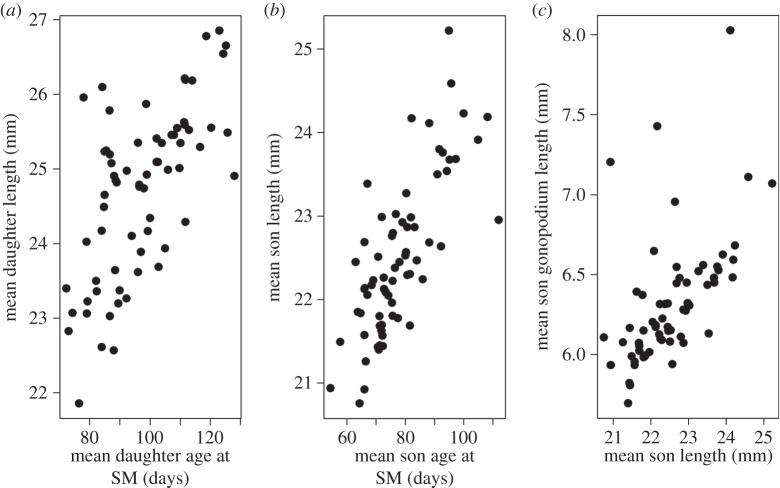
Within-sex associations between maternal effects on different traits. Mean values (raw data) per mother for (*a*) daughters' age and size at sexual maturity (SM); (*b*) sons' age and size at sexual maturity and (*c*) sons' size and gonopodium length. *N* = 69 mothers, 297 daughters and 303 sons.

There was a trade-off between development rate and size at maturity. Thus for both sexes, the phenotypic, maternal and residual covariances between age and size at maturity were all positive (figure 1; electronic supplementary material, table S1; table 1, light grey shading). Maternal effects therefore varied from producing small, fast-developing to large, slow-developing daughters (figure 1*a*). Similarly, sons were either small and fast-developing with a relatively small gonopodium, or large and slow-developing with a relatively large gonopodium (figure 1*b,c*).

**Figure 2.**
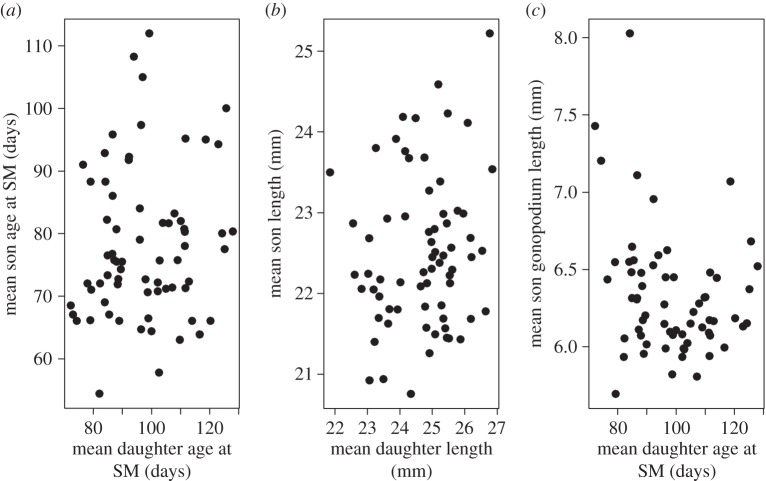
Between-sex associations between maternal effects on different traits. Mean values (raw data) per mother for (*a*) daughters' and sons' age at sexual maturity (SM); (*b*) daughters' and sons' size at sexual maturity and (*c*) daughters' age at maturity and sons' gonopodium size. *N* = 69 mothers, 297 daughters and 303 sons.

Despite the strong correlations *within* each sex, there were no associations between maternal effects on sons and daughters: cross-sex maternal effect correlations were not different from zero (table 1 and figure 2*a,b*). The exception to this was a marginally non-significant (at our critical level of *p* < 0.01) negative correlation between maternal effects on gonopodium size and female development time (correlation = −0.437 ± 0.165 s.e., *p* = 0.016), suggesting that mothers with faster developing daughters had sons with larger gonopodia. However, if the four (of 69) families with largest gonopodia (figure 2*c*) were excluded, the correlation was non-significant (−0.117 ± 0.228 s.e., *p* = 0.588).

## 4. Discussion

Our study of mosquitofish revealed maternal effects that persisted until sexual maturity, and accounted for substantial amounts of the total variance between offspring. This result is consistent with some evidence from other fish species that maternal effects can persist until adulthood [13], although this is not always the case [15]. In a viviparous species such as *G. holbrooki*, and an experimental design in which offspring were raised individually, maternal effects must be generated by pre-natal investment. This investment could be genetically or environmentally determined, though we cannot distinguish the two with our current breeding design. If it is genetically based, maternal effects will have the potential to evolve in response to selection [2]; if, for example, maternal effects on gonopodium length were genetically determined, they could evolve in response to any sexual selection on gonopodium length via sons' reproductive success. The positive association between age and size at maturity (for both sexes, and at both the phenotypic and the maternal effects level) indicates a well-established trade-off: larger body size at maturation takes longer to reach [9]. Development time is presumably under negative selection as, all else being equal, the earlier an individual matures the sooner it can reproduce. This comes at the cost of reduced body size that might lower female fecundity and, depending on the social context, could also reduce male mating success (references in [17]).

Our most important finding was that, despite the significant maternal effects variance on sex-specific traits, there was no evidence for consistent maternal effects across the sexes: what was good for sons was not necessarily good for daughters. A null result might simply reflect low statistical power, but the standard errors on our cross-sex correlations were comparable to those within each sex, and the varying signs of the six correlations indicated no consistent trend (3/6 positive). The only potential exception was the marginally non-significant correlation between maternal effects on sons' gonopodium length and daughters' age at sexual maturity (well-endowed males have fast-developing sisters). We treat this suggestion with caution given its weak statistical support, and its dependence on four families (figure 2*c*), but given potential limitations of statistical power (see below), it may be a result that is worthy of further investigation.

Our results offer a cautionary note regarding analyses of sexually dimorphic traits: had we not split the traits by sex, but had instead considered age or size at maturity as single traits (even if correcting for sex differences in mean values), we would have markedly underestimated the importance of maternal effects as accounting for only 22.7 ± 4.5 s.e.% and 14.5 ± 6.4 s.e.% of the variance in each trait, respectively (compared to 34.9 and 33.7% for the means of sex-specific values, table 1). The underestimation occurs because there is less differentiation between mothers in their average impact on offspring than in their sex-specific effects.

We found no evidence for significant additive genetic variance for any trait. For gonopodium length and female age at maturation (estimates of heritability of 0.166 ± 0.245 s.e. and 0.176 ± 0.140 s.e., respectively), this is probably due to lack of statistical power: simulations [21] indicated low power to detect significant Va with this pedigree and with maternal effect variance components of the magnitude observed (see electronic supplementary material for details), and a recent analysis of gonopodium length in the same study population, using a similar design, observed significant heritability (R. Vega-Trejo 2015, unpublished data). However for the other three traits (body length in both sexes and male age at maturity), the parameter estimates of Va were bound at zero (table 1), giving no indication of genetic variance regardless of statistical power. Excluding maternal effects from the model gave an erroneous impression of significant heritability for all traits (results not shown), confirming that estimates of heritability will be upwardly biased if other sources of covariance between relatives are not taken into account [20].

In sum, investment by mothers into the production of sons versus daughters is well investigated in the context of offspring sex ratios [4], but we know relatively little about subsequent maternal variation in investment into offspring of each sex. Here, we found no evidence of either consistency or trade-offs in sex-specific maternal effects. This suggests independent axes of investment that can follow independent evolutionary trajectories: what defines a high-quality mother depends on the sex of the offspring.

## Supplementary Material

Kruuk Supplementary Information

## Data accessibility

All data are available at: http://dx.doi.org/10.5061/dryad.r2b26.
