# Supplementary material for: Sex-specific maternal effects in a viviparous fish
Source: Biol Lett. 2015 Aug;11(8):20150472. doi: 10.1098/rsbl.2015.0472 (PMC4571680; doi:10.1098/rsbl.2015.0472)
Supplement: Kruuk Supplementary Information [file rsbl20150472supp1.pdf]

Sex-specific maternal effects in a viviparous fish

Loeske E. B. Kruuk, Julianne Livingston, Andrew Kahn and Michael D. Jennions

Supplementary Information

Methods

Fish rearing

We reared mosquitofish (*Gambusia holbrooki*) in our laboratory at the Australian National University. We used a standard full/half-sib breeding design, in which 80 virgin dams were mated to 20 sires, and offspring were then reared individually. Half of the fry from each of the full-sib families were raised in normal growth conditions, whereas the other half experienced an early period of food deprivation. The effects of this food deprivation treatment on juvenile growth rates, compensatory growth and adult traits are reported in [1], where our experimental set-up is described in more detail. Here we recap the main features.

Gambusia females can viably store sperm for several months to produce mixed paternity broods. We therefore only used virgin females in the breeding experiment. First generation lab-born females from the Canberra Lakes stock population (caught in April/May 2010) were raised individually to provide dams for breeding. Sires were randomly selected from the Canberra Lakes stock. Initially, each of 20 sires was mated to four virgin dams (N = 80 pairings). Gravid females were checked daily for offspring. In total, 69 dams produced viable offspring from 19 sires. On the day of birth, up to 10 siblings from each brood were chosen at random, measured, and housed individually in 1 litre aquaria. Up to five offspring from each dam were placed in 'control' and 'treatment' rearing conditions. Offspring in the control group were fed a typical lab diet of brine shrimp *ad libitum* twice daily from birth until the end of the experiment. Offspring in the treatment group were initially fed at the same level for 7 days, after which their feeding was reduced to ~3 mg brine shrimp every second day for 21 days, and then they were returned to normal food levels. In the food-deprivation treatment group, females seemed 'better' than

males at catching up in size without having to delay maturation, suggesting a sex difference in compensatory growth [1]. Each offspring was reared individually, so the only shared environment was due to maternal effects and treatment type. Sickly offspring (identified by external growth of bacteria) and those with spinal curvature were excluded from the study. Partial and full water changes were made as required; excess food was not removed on a daily basis, but the amounts provided were such that food did not build up. There was no excess food during the 7-28 treatment period for the low food group.

The analysis presented here focused on sources of variation in adult traits in N=297 females and 303 males that reached sexual maturity. We defined females as sexually mature by the presence of yellow colouration in their egg sacs, which are visible through the body wall, and males when we could detect a clear apical hook at the gonopodium tip [see 1]. We recorded five adult traits: age (in days) and body length at sexual maturity in each sex, and gonopodium length in males. The standard length (snout to base of the caudal fin, in mm) of each fish was measured from photographs of fish briefly immobilised in cold water. Fish were placed in a Petri dish filled with water, aligned alongside a microscopic ruler (0.1mm gradations), and photographed using a digital camera attached to a dissecting microscope. We used ImageJ software [2] to measure their SL. We re-measured 200 randomly selected photos to confirm that our length measurements were repeatable ($r > 0.95$, $P<0.0001$). Gonopodium length was measured as apical tip to base, in mm, in all males that were alive after 150 days (N = 261 males; we only measured this subset of males due to logistic constraints).

Statistical analyses

We fitted multivariate mixed models to the five traits in ASReml-R [3]. Summary statistics, sample sizes and the phenotypic variance-covariance matrix for the five traits are given in Table S1. All traits were first standardized to unit variance prior to analysis. We fitted *treatment* (as described above, control vs restricted) as a binary fixed effect in all models. *Row* (shelf height in the lab) was also fitted as a fixed effect (a 10-level factor), to account for any spatial variation in

lab conditions associated with this aspect of the spatial configuration of the room. We then quantified components of variance and covariance in the traits using a multivariate “animal model” [4], with random effects of an additive genetic effect (with covariance structure defined by relatedness between individuals) and a maternal effect [grouping individuals by mother, 5].

Because of the food treatment to test for effects of compensatory growth, we also considered a treatment-x-maternal effects interaction, to test whether maternal effects were consistent across treatments. These interactions were never significant ($p>0.20$ for all traits). Similarly, cross-treatment maternal effects correlations were never significantly different from 1. As there was therefore no indication that the maternal effects variance in the restricted food treatment was any different from that in the control treatment, we combined data from the two treatments (and simply included *treatment* as a fixed factor as described above, to correct for any differences in means).

Finally, in addition to the five-trait models of sex-specific traits, we fitted a bivariate model to see what level of maternal effects variance would have been estimated had we ignored the sex-specific nature of the trait variation. This model contained the two traits of length and age at sexual maturity (i.e. not split by sex). Sex was included as a fixed effect (additional to row and treatment) to correct for differences between the trait means; maternal, additive genetic and residual effects were fitted as random effects. As we report in the Discussion, this model returned substantially lower estimates of the maternal effects variance for body length and age at maturity. Adding a sex interaction term to the random effects of this model resulted in a significant improvement to the likelihood ($p<0.001$), further confirming the sex-specific nature of the variance.

Power analyses

We used simulations to investigate the statistical power to detect significant additive genetic variance (Va) in the two traits for which we observed non-zero estimates of Va, gonopodium length and female age at maturity. We used the R package *pedantics* [6] to simulate phenotypes

with our pedigree structure and the observed levels of additive genetic and residual variance (Table 1), and then added a maternal effect common to all offspring of each mother, drawn from a normal distribution, again with the estimate of variance for maternal effects as observed (0.556 and 0.353 respectively for the two traits). We then ran univariate animal models on the simulated data for each trait. The significance of the estimate of additive genetic variance was estimated using a likelihood ratio test of a model fitting Va, Vm and Vr versus one with just Vm and Vr, and the process repeated 1000 times. Estimates of statistical power were calculated as the proportion of runs (out of 1000) for which the p-values were less than 0.01. These were 0.098 and 0.122 for the two traits, i.e. indicating low statistical power to estimate additive genetic variance in the presence of the substantial maternal effects variance observed.

References

[1] Livingston, J.D., Kahn, A.T. & Jennions, M.D. 2014 Sex differences in compensatory and catch-up growth in the mosquitofish *Gambusia holbrooki*. *Evolutionary Ecology* **28**, 687-706. (doi:10.1007/s10682-014-9691-1).

[2] Abramoff, M.D., Magelhaes, P.J. & Ram, S.J. 2004 Image processing with ImageJ. *Biophotonics* **11**, 36–42.

[3] Butler, D.G., Cullis, B.R., Gilmour, A.R. & Gogel, B.J. 2009 ASReml-R reference manual. (http://www.vsni.co.uk/downloads/asreml/release3/asreml-R.pdf.

[4] Wilson, A.J., Reale, D., Clements, M.N., Morrissey, M.M., Postma, E., Walling, C.A., Kruuk, L.E.B. & Nussey, D.H. 2010 An ecologist's guide to the animal model. *Journal of Animal Ecology* **79**, 13-26. (doi:10.1111/j.1365-2656.2009.01639.x).

[5] Kruuk, L.E.B. & Hadfield, J.D. 2007 How to separate genetic and environmental causes of similarity between relatives. *J. Evolutionary Biology* **20**, 1890-1903.

[6] Morrissey, M.B., Wilson, A.J., Pemberton, J.M. & Ferguson, M.M. 2007 A framework for power and sensitivity analyses for quantitative genetic studies of natural populations, and case studies in Soay sheep (*Ovis aries*). *J. Evolutionary Biology* **20**, 2309-2321.

Supplementary Table

Table S1. Summary statistics and overall phenotypic covariances

Summary statistics, sample sizes and the matrix of phenotypic variances and covariances from the multivariate model of length (mm) and age at sexual maturity (age SM, in days) in both sexes and male gonopodium length (mm). Variance estimates on diagonal, covariances below diagonal and correlations above diagonal (all with SEs in brackets). All traits were standardised to unit variance prior to analysis, but estimates of phenotypic variance may be <1 due to fixed effects in the model. Cross-sex covariances/correlations cannot be estimated at the phenotypic level, hence blank cells. P-values are from likelihood ratio tests comparing the full model to one in which the relevant covariance component is constrained to zero.

	body length: male	age SM: male	gonopodium length: male	body length: female	age SM: female
Mean (SD)	22.50 (1.37)	78.17 (18.83)	6.30 (0.47)	24.78 (1.59)	97.78 (19.47)
N	303	303	261	297	297
	length_male	age_male	gonopodium_length_male	length_female	age_female
length: male	0.961 (0.080)	0.786 (0.022) ***	0.682 (0.033) ***	.	.
age SM: male	0.730 (0.069) ***	0.898 (0.074)	0.531 (0.045) ***	.	.
gonopodium	0.663 (0.071) ***	0.499 (0.065) ***	0.984 (0.087) ***	.	.
length: female	.	.	.	0.917 (0.077)	0.700 (0.030) ***
age SM: female	.	.	.	0.674 (0.069) ***	1.010 (0.084)

***p<0.001
